# Cases of Hemorrhage from Wounding the Palmar Arch

**Published:** 1832-04

**Authors:** William Sharp

**Affiliations:** Bradford, Yorks


THE LONDON

### Medical and Physical Journal.

398, VOL. LXVII.] APRIL 1832. [70, *New Series.*

"For many fortunate discoveries in medicine, and for the detection of numerous errors, the world is indebted to the rapid circulation of Monthly Journals; and there never existed any work to which the Faculty, in Europe and America, were under deeper obligations than to the 'Medical and Physical Journal of London,' now forming a long but invaluable series."—RUSH.

### ORIGINAL PAPERS, AND CASES

### OBTAINED FROM PUBLIC INSTITUTIONS AND OTHER AUTHENTIC SOURCES.

### HEMORRHAGE.

*To the Editor of the London Medical and Physical Journal.*

### *Cases of Hemorrhage from wounding the Palmar Arch.*

SIR:

In the last number of the London Medical and Physical Journal, in the Hospital Reports, Mr. Brodie, in speaking of secondary hemorrhage, says, "I was once called to a case of secondary hemorrhage from a wound of the palmar arch, and I cut down upon the humeral artery, and tied it; and I really think this is a better plan than performing two operations for securing the ulnar and radial arteries." I have twice been called to accidents of that kind; once where the superior, and once where the inferior, arch was wounded: in both cases the hemorrhage returned very severely many times, and in each case a cure was effected by tying the radial artery in one case, and the ulnar in the other; so that it would appear that, although the anastomosis of the two arteries is very considerable, yet in accidents of that kind it is not necessary to tie them both.

If you think the above communication worthy of a place in your Journal, it is at your service.

I am, Sir, yours obediently,

WILLIAM SHARP.

*Bradford, Yorks;*
*March 9th,* 1832.

